# Supplementary material for: Patterns of Intron Gain and Loss in Fungi
Source: PLoS Biol. 2004 Nov 30;2(12):e422. doi: 10.1371/journal.pbio.0020422 (PMC532390; doi:10.1371/journal.pbio.0020422)
Supplement: Table S1 — Also available at http://genes.mit.edu/NielsenEtAl/. (4.3 MB ZIP). [file pbio.0020422.st001.zip › NielsenEtAl/html/737.html]

AN4378.1.NCU04310.1.MG01811.1.FG10028.1


```
 CLUSTAL W (1.82) Multiple Sequence Alignments - Introns Inserted


Sequence 1: NCU04310.1	1056 aa
Sequence 2: FG10028.1	956 aa
Sequence 3: MG01811.1	1054 aa
Sequence 4: AN4378.1	885 aa
Alignment Length: 1099 aa
Number Identitical Residues: 241 aa
Alignment Score (without introns) 14293


MG01811.1 	--MQDFTVEVDG-VPDRGPAVFAVTTATLVLATVFVGARLACRGLIVRRFGWDDLSIVIA
NCU04310.1	MEAYSYNHSQDEDVPDRGPAVFVVTTVTICLATLFVVARMVSRIGIIRRVGWDDYIIILA
FG10028.1 	--MSDSASTDDGGIPDRGPVVFAVTTATLVLATVFVVARIISRTFIVRNVTWDDRVMILA
AN4378.1  	-------------MEDRRPEVLVVSIVFFSLATIFVALRFVSRIWVVRRLALHDYLMLLA
          	             : ** * *:.*: . : ***:**  *: .*  ::*..  .*  :::*

MG01811.1 	W~LFAVGISVSIALGTHRGLGRHDENIASEYLPQLRRCEYVFSVLY~NPALMAAKTSVLI
NCU04310.1	W~LISLALSITIDMATRRGLGRHDMDIHPQHRPGLRMCEYVFSILY0NPALMATKTSILV
FG10028.1 	W~LFAFFLSFTICFGVHNGLGRHDENIDPQRLPALRRCEYVFSILY~NPALMATKTSVLI
AN4378.1  	W0LIDLGFSTALFYATKKGLGLHDVDIPVTARSALSSANYAFTVLY0S--------SILV
          	* *: . :* ::  ..:.*** ** :*     . *  .:*.*::** .        *:*:

MG01811.1 	FYLRLSRNTQPLLRLASKIVLFIVNTAGAILTFINIFQCNPINAAYDDVAGQ-----CVP
NCU04310.1	FYLRLGKNTQKVLRMASWAVLVIVNVAGTILTFMNIFQCSPLRAAWDITVES---AKCLP
FG10028.1 	FYLRLAKHTQRVLRFASWLTLAVVNIAGFILTFMNVFQCRPTQAAWDVKYDDRPSAKCIP
AN4378.1  	FYLTLTQ-GEKIFRYANYATLFVVNAAGLALTFVNIFQCRPVEDAFAAQLPAD--AHCTD
          	*** * :  : ::* *.  .* :** **  ***:*:*** * . *:         : *  

MG01811.1 	LLTEFICSAPINILTDLAILALPIPVLTSMRLPPRQKTILVLTFGLGIFVTIVDVVRIYY
NCU04310.1	LLTEFICSAPVNIVTDLAILALPIPVLTGMRLPPRQKTILILTFTLGIFVSIVDVVRIYY
FG10028.1 	LLTEFICSSPVNIVTDLAILALPIPVLTGMRLPSRQKTILVLTFTVGIFVTVVDVVRIYY
AN4378.1  	ILTLYLSSSPVNIITDLAILVLPNPILTRMRLPQKQKIILVVTFSFGFFVAVVDVIRIAY
          	:** ::.*:*:**:******.** *:** **** :** **::** .*:**::***:** *

MG01811.1 	LQNAINYVPSTTQTSDPNAIYGDSPQFSW~SASL0SLMWSAVEVNVGISTACIPTLKPLV
NCU04310.1	LQQAVG-IVSMNFSDDPSAIYGQSVNFSW~NASF~SLMWSAVEVNVGIICACIPTLKPLI
FG10028.1 	LQQAIT-APTS-TSSDPQSRFGGQTDFAW~NASR~SLMWSAVEVNVGMMCACVPTLKPLV
AN4378.1  	LQEATTDREIALRQIHMQN-YGGE-DFAW1YASL~SFMWSVVEVNVSVMCACVPSLKPLV
          	**:*           . .  :* . :*:*  **  *:***.*****.:  **:*:****:

MG01811.1 	LRLLPAMILDPNGNYYGSNNTKDREVLPTAAPAQPAFGAGGGMQAPPPQPSPTSAGNQDQ
NCU04310.1	IRILPAMIIDPDGTRSSTRDRDAAFKRDNSDSSQPVT-----SSTLSDSHASAPQAPEQV
FG10028.1 	LKLLPAMLYDPNGTRASTPK--GPNDSDSPSTGHRAS-----IQQPPPVAQLNAQAPLNA
AN4378.1  	ARLVPKLIRDSSGG----------------------------TQTNPSDPPLPPSGPLQM
          	 :::* :: *..*                              .  .      . .  : 

MG01811.1 	SQQLDTLEFLTTPEMGGTAATPGTPQASNAWSNFRRSTVTTLGAQSSAPTESNLYFGFVS
NCU04310.1	RLNNDRLSDEISIRD---FLAASATNEIPAFEGRTPTFPDRR--RFSSVQENHIYFGFVE
FG10028.1 	NAPMSAMEFLTTPFD---EPAPPRPAQRAAQSNGTYQTSTTM--TSSTGTVEGVYFGFVN
AN4378.1  	QVADAIFSDSLDPRL---TEIATGPTMATTYTDPEANTTTHT---STSDPRSMTFFDFVN
          	      :.             .  .    :  .             ::   .  :*.**.

MG01811.1 	IKRPKSMLKATKAESWRYGAFVTVTFLLWGMSYGLLGTLNNVVAMVADMSVPQTIGLNSI
NCU04310.1	MKKPKSMVKSSVSESLKYCTIVSILFFLWGFSYGLLNTLNNVVANVAHMSTAQALGLTSV
FG10028.1 	MTKPKSMLKCNKKESFKYCTIVSILFLLWGISYGLLNTLNNVIATVDNMTTPETLGMTSV
AN4378.1  	MKKPANMLKLSNKESIAPNAITTVLFFLWGFAYGFLDILNEQFQQIVRLDSWRSLGLHCV
          	:.:* .*:* .  **    ::.:: *:***::**:*. **: .  :  :   .::*: .:

MG01811.1 	YFGLGYLLGPLIVGVWILRHDEHHRSRHATRNHRPHESIGGFKATLMLGLAIFGTGTICF
NCU04310.1	YFGGGYLLGPLLVGGWLLCHDEHRRFRRRRRGD--IEPIGGFKATFIVGMLIYGVGTIMF
FG10028.1 	YFGGGYFFGPLLVGEWILRRDEHNRSKRHKGNG--HDSVGGFKVTFIVGLCFYGIGTVIF
AN4378.1  	YFG-GYLVAPPLVGRPVLKH-------------------WGFKSTFITGLCFYACGSLIF
          	*** **:..* :**  :* :                    *** *:: *: ::. *:: *

MG01811.1 	WPAAVLASFPGFMVSNFI1VGSGLAVLETAANPFLVLCGPSEYATMRLCLAQGVQAVGSV
NCU04310.1	WPCAVLSAYGGFMASSFV~VGFGLAVLETAANPFLVLCGPPEYADMRLLLAQGVQAVGAV
FG10028.1 	WPSAVTQSYAGFMLSNFV~VGFGLSILEVGANAFLILCGPPEYGETRVLMAQGVQGIGSV
AN4378.1  	WPSAVLTSYSAFLVSNFI~TGCGLAILETAANPFISLCGPLENSEVRLNISQGVQAVGSV
          	**.**  :: .*: *.*: .* **::**..**.*: **** * .  *: ::****.:*:*

MG01811.1 	LSGLLAQHVYFSNISQRQERPDSLILSDVQWTYLAVTLFCAALALLFYYIPLPEVSDQEL
NCU04310.1	LSGVLANNVFFHKIEERK--HSPTTLLDVQWTYLTITLLCVLLALFFYYSPLPEVTDREL
FG10028.1 	LSGLLAQKVFFKGIVEQNN-ASSMTLINVQWTYLGITLLCVALGLFFYYMPLPEVSDREL
AN4378.1  	LSPLLAKKVLFRSVTDVS------SLVDVQWTYLAIALFDVLLALAFYYLPIPEASDEDL
          	** :**::* *  : : .       * :****** ::*: . *.* *** *:**.:*.:*

MG01811.1 	AASAEKLPVDSTKRSIGGLQLRTVCIILAVLAQYCLIAAQESVSIFFQDLVSALVPTESA
NCU04310.1	GRLSERLPVEPKKRSIGGVSLRTWCILLAVFSQWFYVAAQENMSVFFTRLMTTFSPTH--
FG10028.1 	EQSATHLPMDPQKKRSG-LQLRTWSLILAVLAQYTYVAAQECFSIYFRSLMISILPNQPN
AN4378.1  	EELANRRRDDNRTAPFG-IPVVWMTLALGIWSQFFYVAGQEVFSTGFGALIATAYADP--
          	   : :   :  .   * : :    : *.: :*:  :*.** .*  *  *: :  .    

MG01811.1 	TPIASVSSGTGSGSTANATNSEPNPPSLTLSVSNYVVISHTAFAISRFLAGGLAYLSAKH
NCU04310.1	---------EPYPNS--------RPEGFEIRTLNYLVVSHTAFAISRFIAAYLCYLHVKR
FG10028.1 	TGEEAAQGASDIPNTD-------KPPGISLSIPDYLLIAHTAFTVSRFMAAGLTYLSVYR
AN4378.1  	----------------------------PLSEFTYLTIGRAIFAVGRFLAAFL-------
          	                             :    *: :.:: *::.**:*. *       

MG01811.1 	P-RNKWIPTPRTVLLTSIFTAVMAMIAIITVKRSSSLIGPNIIAIPIVIFFFAEGPGWPL
NCU04310.1	GPTSRFIPTPRTILSICAALATVFILVTVVYKPAS---NPNLMAIPIILFFFFEGPIWPL
FG10028.1 	---SRFP-RPRTILTACIAGCFLFALLPVVVRPP----NPNLLIIPIVLFYFFEGPIYPL
AN4378.1  	----QWFLKPRWILLLSYIGMVVFAALCMHTTGAA------AIASAMMVFLFESG-TFSI
          	    ::   ** :*  .     :     :    .:       :  .:::* * .*  :.:

MG01811.1 	IFSLGLVGQGSRTKLAAAWLTMGASGPAFVPFIIYAIVQRSDTGRNIQVAFCVVAAVLCF
NCU04310.1	IFSLGMRGQGKRTKQTAAWLTMGGSGPAFWPFVSYAILQR---GASIQTSFIVVVILMAM
FG10028.1 	VFAIGLRGQGRRTKRAAAFITMGSSGPGFFPFVMYGIIKH---GGTVKTAFIVVVALQAV
AN4378.1  	IFAIALRGTGRHTKTAASLLTIAISGGAFLPFARYGAQLA---ANSTLDSYSVLVALFAA
          	:*::.: * * :** :*: :*:. ** .* **  *.       . .   :: *:. : . 

MG01811.1 	TLVYPLYLCFGRDARKM----VDPVDP-RDQPAMPSP--------------------AEE
NCU04310.1	TMVYPLFLTFVRDAREMSDHVMDTAAQKRRRSGDVEDGGGGLGGLGGRGVFLSERRGSEN
FG10028.1 	AMSYSIFLQSVRDVKRM----VDPCPAVRDALGQPDE---------------------EM
AN4378.1  	GALFPVYLNLVPAAKKQ----VDPVPN---------------------------------
          	   :.::*     .:.     :*.                                    

MG01811.1 	QVGIDTLLERRMRGR----LSAMSAEGRGTSKSTLANTNDWEEDGPRSIALFDKLKNGLE
NCU04310.1	PMTLDQIIAQRQRERVKAAQKAARAENGGMLKRLSRSMGIGTEETPDENIKDEKRRSRQQ
FG10028.1 	PSPMETVMADRARR-----HSTIRAKSPGIFGKVSQST-----RVIGAKFLSSRRRSSQ-
AN4378.1  	----EHLRHTRRRSR-----RQGRAQAANVLPSEKANP--------SEGGVLSRQRSVLD
          	    : :   * *           *:  .       .               .: :.  .

MG01811.1 	AAKQGLHEHHTNSPLKGSRNNSPRRPSATSTSPR----SAVSTLRGERLETTDEIKEEEE
NCU04310.1	QQQGQGQMQQHLQPEMQYEKQSGKKTSGEDEPPRKEGESDEDPMSGADEARHPNPPAPET
FG10028.1 	-------------PSIQQCEDS----------------RDNVPL----------------
AN4378.1  	DADPLPSVDFSSPNSVASRSRS----------------DSAESDSGADADSGADADVVDF
          	  .                . *                    .  .       .    . 

MG01811.1 	VGEERENRSSGEDGEKLKPPF---
NCU04310.1	PEWERQTAPWDLPQLQLDTRILED
FG10028.1 	------------------------
AN4378.1  	QCTRTKAVSSTSDEMSRT------
          	     .  .      .
```
